# Intracranial Recordings in Epilepsy: an Approach to Understanding the Neural Mechanisms of Social Cognition

**DOI:** 10.1007/s11910-025-01431-9

**Published:** 2025-06-28

**Authors:** Kaitlyn E. Davis, Rajesh K. Kana, Jerzy P. Szaflarski

**Affiliations:** 1 Department of Neurology, UAB Epilepsy Center, University of Alabama at Birmingham Heersink School of Medicine, 1720 7Th Ave South, Birmingham, AL 35294 USA; 2 Department of Psychology, University of Alabama at Birmingham, 1720 7Th Ave South, SC 613, Birmingham, AL 35294 USA

**Keywords:** Intracranial EEG, Direct electrical stimulation, Brain mapping, Epilepsy, Social cognition, Theory of mind

## Abstract

**Purpose of Review:**

Social cognition, especially theory of mind (ToM), is often impaired in clinical populations including epilepsy, with serious consequences for quality of life. This review synthesizes evidence from clinical assessments, neuroimaging, and intracranial recording techniques to examine how social cognitive processes are represented in the human brain and how they may be disrupted in epilepsy. Emphasis is placed on the emerging contributions of intracranial electroencephalography (iEEG) and intracranial electrical stimulation (iES) to this field.

**Recent Findings:**

While the literature using iEEG/iES to investigate social cognition remains limited, recent studies have begun to map the spatiotemporal and causal underpinnings of ToM and related functions. This work complements behavioral and fMRI findings and suggests avenues for translational research.

**Summary:**

Intracranial techniques represent a promising tool for advancing social cognitive neuroscience. Integrating this approach with clinical data and established behavioral paradigms can enhance our understanding of social cognition and improve care for populations with focal epilepsy.

**Supplementary Information:**

The online version contains supplementary material available at 10.1007/s11910-025-01431-9.

## Introduction

**Fig. 1:**
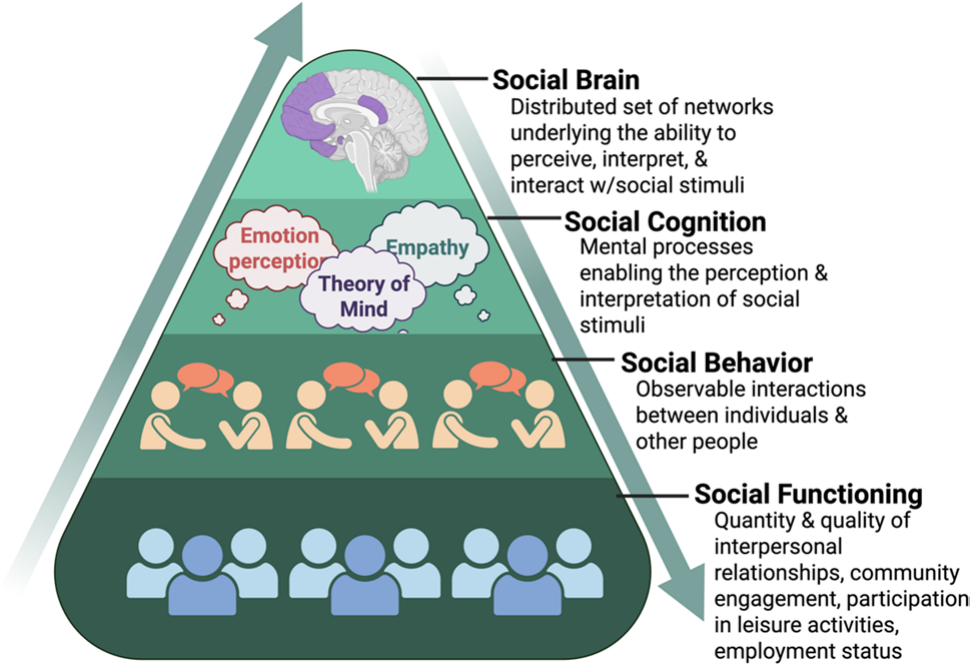
Schematic of social levels, Social brain networks enable social cognition & behavior, ultimately influencing social functioning. Figure based on Kennedy & Adolphs (2012) & created in BioRender.com

The human experience is fundamentally shaped by our ability to form and sustain social relationships, a process that hinges on correctly interpreting and responding to social cues. Failures in this ability can severely disrupt social communication and are a feature of many neuropsychiatric disorders including epilepsy [1–4]. Processing social stimuli relies on a set of cognitive functions collectively known as social cognition. Social cognition refers to the capacity to identify, interpret, and respond to socially relevant information and depends on neural systems that process the perception of social signals and link them to motivational, emotional, and behavioral responses [5]. It encompasses basic abilities such as face processing and more complex functions such as theory of mind (ToM) [6]. ToM is considered a particularly important aspect of social cognition and is defined as the ability to infer and predict the unobservable mental states of others, such as their beliefs, desires, emotions, and intentions [7]. Rather than solely reacting to the observable actions of others, ToM allows us to anticipate their behavior based on those inferred mental states and to adapt our responses accordingly—helping us to recognize social cues and navigate the social world smoothly [8]. ToM deficits are the key characteristic of autism spectrum disorder (ASD) and a feature of many other conditions including schizophrenia [9], Alzheimer’s disease [10], and epilepsy [11], with a significant impact on social functioning and overall quality of life (QoL) [3, 9, 12, 13]. As neuroscience has shifted toward a network-level view of the brain, the understanding of social cognitive deficits has also evolved. Social cognitive dysfunction is now seen as arising from abnormal interactions within distributed social cognition networks rather than isolated disruptions in specific brain regions with a significant downstream impact on social functioning (Fig. 1) [4]. Most of what is known about these brain networks is derived from functional magnetic resonance imaging (fMRI) studies [14], but fMRI’s limited temporal resolution and correlational nature preclude the measurement of rapid interactions and causal relationships [15]. As a result, it is unclear how the networks identified by fMRI interact to produce social cognitive functions including ToM or what brain networks are necessary for these abilities.

**Fig. 2:**
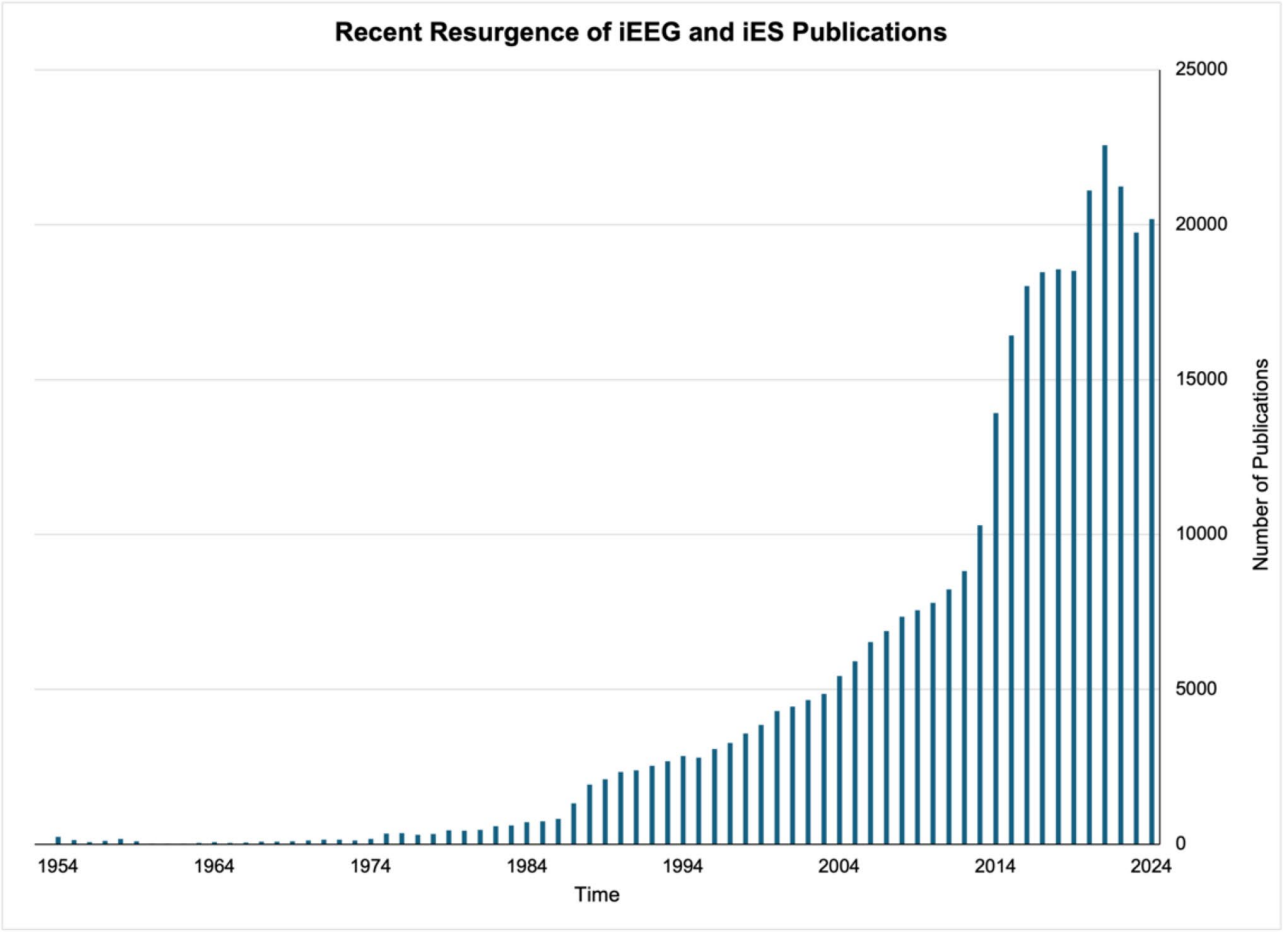
Number of publications in PubMed using the terms: "iEEG", "intracranial electroencephalography", "sEEG", "stereoelectroencephalography", "ECoG", "electroencephalography", "iES", "intracranial electrical stimulation", "DES", or "direct electrical stimulation"

Increasingly, researchers are harnessing intracranial electroencephalography (iEEG) and intracranial electrical stimulation (iES) to account for the limitations of functional neuroimaging methods (Fig. 2). These techniques are clinical tools that involve the implantation of electrodes in patients undergoing pre-surgical evaluation for the treatment of focal drug-resistant epilepsy (DRE). However, patients are often recruited to participate in research during their hospital stay as iEEG and iES also offer a unique window into the neural mechanisms of human cognition and behavior. Using these techniques to better understand the neural mechanisms of socio-emotional processes is an emerging research area [16, 17]. The purpose of this review is to examine how iEEG and iES can expand our knowledge of the neural mechanisms underlying social cognition and its disruption in clinical populations to extend prior fMRI findings and inform targeted interventions. We focus on ToM and its disruption in focal epilepsy. We begin with an overview of using these techniques in clinical and research settings and then discuss the few studies that have leveraged them to characterize social cognition networks. Next, we situate social cognition within a clinical context and describe common behavioral assessments of ToM in focal epilepsy and their psychometric properties. We conclude by proposing an approach and future directions for iEEG/iES research in social cognition to advance our understanding of how the brain supports social cognitive functions such as ToM and to enhance the clinical relevance of findings, particularly for focal epilepsy.

## Methods

For this narrative review, we conducted a literature search in PubMed using combinations of the following terms: “intracranial EEG”, “electrocorticography”, “stereo-EEG”, “direct electrical stimulation”, “intracranial electrical stimulation”, “social cognition”, “theory of mind”, “mentalizing”, “mindreading”, “empathy”, “epilepsy”, and “drug resistant epilepsy”. We included original research articles published in English that used intracranial methods (iEEG and iES) to investigate social cognition in humans, with special attention given to ToM. Due to the small number of such studies currently available, we included all articles published within the last 15 years from 2010 to 2025 and incorporated relevant clinical, behavioral, and neuroimaging literature.

## Overview of Intracranial Monitoring

### Intracranial EEG Monitoring as a Clinical Technique

IEEG is an important tool in the clinical evaluation of patients with focal drug-resistant epilepsy (DRE). When traditional medications fail to adequately control seizures, iEEG is used to localize the brain region responsible for seizure propagation, i.e., the seizure onset zone (SOZ), and to map critical functional regions. This information guides personalized treatment strategies, such as resective surgery, responsive neurostimulation (RNS), or deep brain stimulation (DBS). Before a surgical or neuromodulatory intervention is selected, patients undergo presurgical evaluation using iEEG which records the brain’s electrical activity from electrodes placed directly on or within the brain [18]. The implanted electrodes capture neuronal activity at millisecond (ms) and millimeter (mm) scales providing vital information about the epileptic network’s location and functional impact with temporal and spatial precision. During the presurgical evaluation, patients also become potential research participants as they offer a rich source of information that is otherwise not available. There are two forms of iEEG which use two different types of electrodes and are sometimes combined. Electrocorticography (ECoG) implants subdural electrode grids on the surface of the brain to record from cortical areas and stereoelectroencephalography (sEEG) implants depth electrodes within the brain to target subcortical structures (e.g., amygdala). Once the electrodes are implanted, patients are weaned off their medications and the recordings are monitored for seizure activity until enough data is collected to identify the SOZ. In addition to passively recording neural activity, the presurgical evaluation involves delivering intracranial electrical stimulation (iES) through implanted electrodes to reproduce seizures and symptomology and to map vital functions (eloquent cortex) [19]. The current review focuses only on the latter when discussing iES. The goal of mapping cortical functions is to preserve brain areas from surgical resection that are critical for important abilities, e.g., language and motor function. IES involves applying stimulation directly to brain areas, typically to the cerebral cortex, while patients perform behavioral tasks. If the behavior is disrupted repeatedly during stimulations, the region is considered critical for the cognitive process being tested (i.e., eloquent cortex). IES can be performed intra- or extra-operatively and is also used to map eloquent cortex for tumor resections [20]. Currently, there is no standardized stimulation protocol, but disrupting behavior typically involves the following stimulation parameters: 50 Hz frequency, 0.5–1 s (s) pulse width, 0.5–5 amplitude (mA), and 3–8 s duration [21].

### Intracranial EEG Monitoring as a Research Tool

While iEEG and iES are vital components of surgical planning in epilepsy, they also provide substantial insights into the functional organization of the human brain. In the 1950 s, Dr. Wilder Penfield used iES in the 1950 s to map the sensory and motor cortices in epilepsy patients [22] and more recently, this technique has been employed to reveal the distributed organization of language networks [23]. Today, there is a resurgence of interest in using iEEG and iES as research tools in cognitive neuroscience to bridge gaps left by fMRI and other modalities [24, 25]. For over 30 years, human brain mapping research has relied on correlative neuroimaging techniques to map cognitive processes particularly, fMRI [26]. FMRI’s ability to provide whole brain scans with high spatial resolution has enabled evaluation of large-scale neural networks, but the blood oxygen level dependent (BOLD) response remains an indirect measure of neural activity with a relatively poor temporal resolution [15]. As a result, the brain regions necessary for social cognitive processes including ToM and their interactions remain largely unexplored. Moreover, findings from fMRI research have not been routinely translated into therapeutic targets for brain disorders, leading to a debate over the clinical utility of functional neuroimaging methods as they cannot distinguish between brain regions that are causing a symptom or behavior from those correlated with it [24, 27, 28]. In addition, fMRI’s low temporal resolution (typically 1–3 s) [29] prevents the measurement of rapid neural responses and directional relationships [30, 31]. Consequently, the network interactions producing many cognitive processes remain unclear.

In contrast, iEEG’s high spatiotemporal resolution can capture millisecond-scale neural activity with anatomical precision to effectively map the direction of information flow within social cognition networks [32–34], while iES can transiently disrupt network function and behavior to identify the brain regions necessary for specific social cognitive functions [35, 36]. Neural communication within social cognition networks can be further characterized by leveraging the high temporal resolution of iEEG which enables the measurement of oscillatory activity in a wide range of frequency bands, from delta (1–3 Hz) to low gamma (30–50 Hz) [32, 34, 37]. IEEG recordings also permit the assessment of higher frequencies during social cognitive processing, referred to as high gamma or high-frequency broadband (HFB) activity, which typically fall within 50 to 180 Hz [32–34]. HFB activity is considered a measure of local neuronal activity and is often used as a point of comparison with fMRI, as it correlates with rapid neuronal firing and the BOLD signal [15, 38]. It is time locked to the presentation of a specific stimulus, making it different from the pathological high frequency oscillations found in epilepsy, which are often coupled with interictal epileptiform discharges [25]. Employing iEEG and iES for research purposes involves recruiting neurosurgical patients to participate in studies during their hospital stay and in the case of iES, collaborating with an epileptologist and/or neurosurgeon to administer and monitor the effects of stimulation on participants. Stimulation parameters for iES research are typically the same or similar as those used in clinical settings to map cortical functions (see previous section). Overall, iEEG and iES serve as both important clinical techniques in neurosurgery and as research tools in cognitive neuroscience, with potential to extend the current mechanistic understanding of social cognition beyond what is possible with fMRI.

### Limitations

IEEG/iES studies are limited by several methodological constraints. Due to its invasive and clinical nature, this research is only conducted in patient populations and sample sizes are usually small restricting the generalizability of findings. Additionally, the anatomical placement of electrodes is determined by the patient’s medical team and is based on their clinical hypothesis of the SOZ location, resulting in variable and incomplete brain coverage across participants. However, several factors and research methods enhance the interpretability and relevance of iEEG/iES findings for healthy brain function. While their location is dictated by clinical needs, electrode implantations follow a consistent pattern. Most are implanted in limbic and frontal regions as the majority of patients are suspected of either temporal or frontal lobe epilepsy (TLE/FLE) [25], and therefore, will overlap with social cognition networks. Moreover, most electrodes are not located in the epileptogenic network providing recordings from non-pathological tissue [25]. Current approaches to conducting iEEG/iES research reduce the influence of pathological activity by applying rigorous data cleaning techniques to remove epileptiform activity and general cognitive screening to exclude patients with substantial deficits [39, 40].

## Adding the Temporal Dimension to Social Cognition Networks using iEEG

Extensive fMRI research has unveiled the distributed brain networks selectively active during social cognition tasks. These studies consistently implicate the temporo-parietal junction (TPJ), medial prefrontal cortex (mPFC), inferior frontal gyrus (IFG), and precuneus in ToM functioning [41, 42]. Passive iEEG recordings can extend the fMRI literature by providing detailed spatiotemporal descriptions of neuronal activity at millisecond and millimeter scales, enabling the assessment of network interactions at the speed of cognition. A recent iEEG study uncovered, for the first time, the precise spatiotemporal dynamics of ToM. Researchers explored potential differences in thinking of oneself vs others—the self-other distinction is a key facet of ToM and represents an important milestone in human development [43, 44]. IEEG recordings were collected from 16 patients undergoing presurgical evaluation for DRE while they judged the personality traits of themselves and others (e.g., “I am honest.”, “My neighbor is honest.”) [33]. Findings revealed a shared pattern of HFB activity (70–180 Hz) within default mode network (DMN) regions, beginning in the visual cortex (~ 100 ms), progressing through temporo-parietal areas (~ 300 ms), and culminating in medial prefrontal regions (~ 460 ms) [33]. In contrast, DMN regions appear to respond simultaneously when viewed through the lens of fMRI which is clouded by a seconds-long temporal resolution. Compared to a math control condition, later-stage regions exhibited stronger functional specificity for the trait judgment task and were more predictive of behavioral responses, suggesting a hierarchical processing model in which initial stages occur in temporo-parietal regions before advancing to the mPFC for higher-level ToM processing [33]. Notably, the study identified robust differences in the duration of self-other responses with later-stage regions, including the dorsomedial PFC (dmPFC), displaying slower more prolonged activation during other-judgements [33]. This result indicates that considering the states and traits of other people requires more effortful processing than thinking of oneself and contradicts extant fMRI findings of the dmPFC as ‘other-selective’ [33]. FMRI studies consistently report preferential responses in the dmPFC to thinking of others compared to oneself [45] with the standard assumption that activation differences reflect variance in the magnitude/strength of responses [33]. However, self-other judgment recruited identical dmPFC sites with no difference in response magnitude, leading researchers to propose an alternate mechanistic account of hemodynamic responses—one that depends on activation duration rather than activation intensity [33]. Together, these findings make important contributions to understanding the neural mechanisms of ToM by revealing a spatiotemporal sequence and functional gradient of activation for this process that is obscured in standard fMRI studies.

Similar progress has been made in understanding the spatiotemporal dynamics and connectivity patterns of empathy. Empathy is an aspect of social cognition that enables us to share the emotions of others [46] and is important for prosocial behavior [47]. FMRI studies have pinpointed the brain regions involved in this process to include the anterior insula (AI), anterior cingulate cortex (ACC), amygdala, and inferior frontal gyrus (IFG) [48]. Recent iEEG findings characterized the temporal and spectral patterns of neural activity within this network while 22 neurosurgical patients with DRE viewed images of others experiencing a painful vs non-painful stimulus [37], a common paradigm used in empathy research [49]. The perception of others’ physical pain evoked a rapid cascade of neural activity in various frequency bands. First, HFB activity (70–150 Hz) was detected in the IFG followed by altered beta responses (15–35 Hz) in the AI (~ 120 ms), ACC (~ 180 ms), and amygdala (~ 370 ms) [37]. The coordination of such oscillatory activity is thought to facilitate communication between disparate brain regions [50], particularly through low-frequency coupling—synchronized activity in lower-frequency bands which is considered to support long-range communication [51] and phase-amplitude coupling, in which the phase of low-frequency oscillations modulates the amplitude of high-frequency activity [52]. Researchers assessed these measures of functional connectivity within the empathy network during vicarious pain perception [37]. Findings revealed low-frequency coupling between the AI, ACC, and amygdala within the beta frequency band and phase-amplitude coupling between these regions and the IFG such that the beta oscillations modulated the IFG’s high gamma response [37]. The results from this study provide a spectral-temporal-spatial map of neuronal activity and inter-regional communication during empathic pain responses to uncover the rapid, coordinated processes underlying our ability to understand others'suffering. Together with the ToM findings described earlier, these studies demonstrate how iEEG can reveal the millisecond-scale neural dynamics underlying complex social cognitive functions with anatomical precision to complement and extend previous fMRI findings. that appear simultaneous in neuroimaging studies with lower temporal resolution.

## Causally Connecting Social Cognition to Brain Regions with iES

Equipped with the ability to directly record brain activity and transiently disrupt it through focal stimulation, iES is the most precise causal human brain mapping technique [24]. IES can be applied directly to brain regions to temporarily disrupt performance on social cognition tasks revealing necessary nodes for these functions. Mapping socioemotional functions with iES is still nascent [16, 17], but ToM is one of the few processes that have been investigated in this research area. These studies have been conducted intraoperatively in patients undergoing awake surgery to remove right-hemisphere gliomas using the Reading the Mind in the Eyes task (RMET) [35, 36]. The RMET involves identifying mental states from grayscale images of eye regions [53]. In an initial study with five patients, researchers linked ToM deficits to the right inferior frontal gyrus (IFG), specifically the pars opercularis and pars triangularis [36]. Stimulating these areas impaired RMET performance, causing participants to select the wrong mental state [36]. Axonal connectivity analysis in two patients revealed crucial sites in the white matter connectivity of the pars opercularis [36]. These findings suggest that the right IFG and its white matter connections are crucial for understanding the minds of others. A follow-up study in 27 patients expanded these findings, mapping additional regions and pathways [35]. In addition to the IFG, stimulating the posterior dorsolateral prefrontal cortex (dlPFC), posterior superior temporal gyrus (pSTG), and TPJ also impaired RMET performance [35]. White matter analysis pinpointed critical sites within the inferior fronto-occipital fasciculus, the white matter pathway connecting the occipital cortex with orbito-frontal regions, and the superior longitudinal fasciculus/arcuate fasciculus (SLF/AF) [35]. The SLF is a major white matter tract linking areas in the occipital, frontal, parietal, and temporal lobes and encompasses the AF, a pathway connecting language regions in the frontal and temporal lobes [54, 55]. Importantly, disruptions in these areas did not affect semantic memory, decision-making, spatial cognition, and language skills, demonstrating their role was specific to ToM [35]. Overall, despite the limited number of studies, iES findings of critical sites for ToM functioning confirmed the importance of the IFG, dlPFC, STG, and TPJ, corroborating the network identified in prior fMRI research using the RMET [41]. Moreover, iES pinpointed critical areas within major white matter pathways, extending previous fMRI findings by revealing the importance of long-range connectivity to inferring the mental states of other people.

## Clinical Implications of iEEG/iES Investigations of Social Cognition

iEEG/iES studies comprise an exciting and relatively new area of cognitive neuroscience research with important clinical implications. The combination of high temporal and spatial resolutions plus the ability to manipulate brain function with focal stimulation has enabled researchers to make new discoveries about the networks localized in fMRI investigations of social cognition [33, 35–37]. The ability to precisely identify when and where a cognitive process like ToM occurs could help reveal the specific timing and location of social cognitive deficits, e.g., whether impairments emerge during early processing stages and reflect disruptions in prefrontal connectivity. Such information is valuable for determining when and where to deliver targeted treatment interventions and for identifying regions critical to social cognition to potentially avoid during surgical resections. Incorporating the frequency dimension provides an additional layer of information by characterizing how brain networks interact during social cognitive processes. These neuronal oscillations, or brain rhythms, are a major mechanism of brain function and their coordination across widespread networks is thought to form the neurophysiological basis of cognition [50]. As iEEG/iES research has shifted from investigations of isolated brain regions to analyses of broader networks, studies have begun to uncover distinct frequency-specific patterns of communication, including low-frequency synchrony [32, 34, 37] and phase-amplitude coupling between regions during socioemotional processing [34, 37]. These findings offer important clues into how network interactions may be disrupted during social cognitive dysfunction, particularly in epilepsy, where abnormal oscillatory activity is a hallmark of the disorder [56] and has been linked to cognitive deficits [57]. Characterizing the frequency content of functional networks could also inform the development of neuromodulation techniques, such as responsive neurostimulation (RNS), by identifying frequency-specific biomarkers of dysfunction and potential targets for therapeutic intervention.

## Current Clinical Perspectives on Social Cognition and Common Assessments of ToM

Social cognitive deficits are transdiagnostic with evidence indicating that processes such as ToM and facial emotion recognition are impaired in most neuropsychiatric disorders [11]. While social cognition overlaps with general cognitive abilities [58, 59], research has shown these domains are dissociable, demonstrating that they rely on distinct neural systems [60, 61] and that social cognitive deficits can exist in patients with otherwise intact cognitive and perceptual abilities [62–65]. This distinction is now formally recognized in the Diagnostic and Statistical Manual for Mental Disorders (DSM-5), which lists social cognition as an essential neurocognitive capacity, alongside memory and executive control [66]. Moreover, social cognitive dysfunction can have a distinct impact on wellbeing. Studies examining this relationship in epilepsy found ToM performance was related to overall QoL and social functioning, while other cognitive abilities (e.g., executive control) and clinical variables (e.g., seizure frequency) were not [2, 3, 67]. Similar findings have been reported in autism spectrum disorder (ASD), Alzheimer’s disease, and schizophrenia [12, 13, 68, 69]. Given its potential clinical significance, there is a growing push to include assessments of social cognition in neuropsychological evaluations [11, 70–73]. While social cognitive abilities are not routinely tested in the examination of epilepsy, they are increasingly recognized as important [74, 75], and recent guidelines recommend assessing emotion recognition, ToM, empathy, and social behavior when social difficulties are suspected in patients [72]. In contrast, others recommend testing only ToM and emotion recognition, the most established deficits in epilepsy, as comprehensive assessments may be impractical in clinical settings [73]. The importance of preserving social cognitive functions in neurosurgical patients is also gaining recognition [16, 17]. The clinical goals of maintaining QoL in neurosurgery have evolved from only preserving language and motor function from surgical resection to also protecting socioemotional processes [16, 17]. Though the assessment of social cognition is not yet standard practice in clinical settings, many of these tasks have demonstrated strong specificity for detecting deficits in neuropsychiatric conditions [70, 76]. In the next section, we discuss a few of the most validated ToM assessments with a focus on their use in focal epilepsy, neurosurgery, and fMRI.

### Reading the Mind in the Eyes Task (RMET)

Originally developed to differentiate ToM from general intelligence in adults with ASD, the RMET is designed to distinguish ToM processes from other central mechanisms by reducing executive functioning and language demands [53]. The RMET consists of 36 pictures depicting only the eye region of the face presented sequentially and requires participants to choose one of four emotions that best describes the person’s emotional state [53]. The control condition often involves determining a non-mental aspect of the photograph (e.g., biological sex). Contrasting these conditions in fMRI studies consistently reveals increased activation in the IFG, TPJ, and middle cingulate gyrus in response to mental state judgements over non-mental judgements [41]. The RMET has demonstrated good test–retest reliability and convergent and discriminant validity [77]. In addition, it has shown clinical sensitivity and specificity for detecting ToM impairments in neurological disorders [70]. A study in frontal lobe epilepsy (FLE) found patients scored significantly worse on the RMET and other affective ToM tasks compared to healthy controls, with performance 2 to 3 standard deviations (SD) below the control mean—indicating a moderate to large effect size [78]. These deficits were not correlated with deficits found in executive functioning which were mild in magnitude (1–2 SD below the control mean) [78]. Conversely, patients exhibited intact performance on tasks that required inferring non-emotional mental states, suggesting FLE may distinctly impair the ability to understand the emotions of others [78] and demonstrating the RMET’s ability to detect specific ToM deficits in patients with focal epilepsy. The RMET has also demonstrated clinical sensitivity for detecting ToM impairment following neurosurgery. Performance on the this task significantly declined after resection of the pars opercularis in patients undergoing tumor surgery [79]. The RMET has since become one of the only social cognition tasks adapted for use in a neurosurgical setting [16]. Together, these findings underscore the RMET’s specificity for isolating affective ToM processes and their neural correlates as well as its sensitivity to social cognitive impairments in clinical populations, making it a valuable tool for characterizing focal epilepsy-related deficits and tracking postoperative changes.

### False Belief Task

Born from the field of developmental psychology, the False Belief Task is the oldest formal test of ToM [43, 65]. It consists of 40 vignettes describing a character’s false belief about a situation (ToM condition) contrasted with descriptions of outdated photographs (Control condition) [80, 81]. Participants must read each vignette and answer a true/false question about it. The ToM condition requires participants to set aside their own knowledge of the situation and adopt the character’s perspective to understand the character is holding a belief that contradicts reality. This self-other distinction helps control for the potential confound of projecting one’s own mental state onto others. Similarly, the Control condition requires participants to separate a photo’s outdated depiction of a scene from what the scene looks like currently. Thus, the stories are matched on their structure, inaccurate content, and cognitive demands but differ in whether they require representing a person or an object. Contrasting these conditions in fMRI studies of healthy individuals consistently reveals preferential responses in the TPJ and dmPFC to the ToM condition (false belief stories) [14, 41, 42, 61, 82], even at the individual trial level [61]. While the False Belief Task is one of the most common ToM assessments used in fMRI research [14], it is less frequently employed in the study of epilepsy [83]. Research using this task has demonstrated that patients with TLE perform worse than healthy controls, particularly those with right or bilateral TLE, with a large effect size (eta^2^ = 0.24), indicating that group membership explains approximately a quarter of the variance in false belief reasoning performance [84]. In contrast, these findings showed no difference in performance on other cognitive assessments except for tests of memory function which were not correlated with scores on the False Belief Task [84].

### Faux Pas Test

The Faux Pas Test was designed to detect subtle ToM difficulties in clinical populations [85–87] and is the most common ToM assessment in epilepsy [72, 83, 88]. It consists of vignettes describing situations in which a character says something socially inappropriate without realizing it, contrasted with stories that do not contain a faux pas [89]. After the story is presented, participants are asked whether a faux pas occurred. If they answer yes, additional questions are presented to interrogate participants’ understanding of social norms and etiquette (e.g., “why shouldn’t the speaker have made that comment?”) [89]. The task is considered a more complex measure of ToM because it involves representing two mental states: understanding that the speaker is unaware they said something inappropriate and that the recipient might feel insulted or hurt. People with focal epilepsy consistently exhibit diminished performance on the Faux Pas Test compared to healthy individuals [3, 90–92]. For instance, findings have shown patients with right, left, and bilateral TLE performed worse on this task compared to healthy controls with a large effect size (eta^2^ = 0.48), indicating that group membership explains almost half of the variance in Faux Pas Test performance [84]. Moreover, impaired performance on the Faux Pas Test has been shown to predict poor social functioning (e.g., unemployment and difficulty maintaining interpersonal relationships) in surgical candidates with TLE over other ToM assessments, including the False Belief Task [2]. These findings also demonstrated Faux Pas Test scores predicted social functioning better than measures of other cognitive functions including memory and executive function [2]. The Faux Pas Test has been employed to evaluate ToM functioning after TLE surgery, but several studies have reported no difference between pre-operative and post-operative performance, suggesting anterior temporal lobectomies do not alter this ability [93–95]. Although this task is widely used to assess ToM in clinical research, relatively few studies have employed it in conjunction with fMRI. As a result, the neural mechanisms underlying faux pas detection remain less extensively characterized compared to other ToM tasks, such as false belief paradigms. This gap highlights the need for future neuroimaging research to better elucidate the brain networks involved in complex social reasoning processes assessed by the Faux Pas Test.

## Using Intracranial Techniques to Study Social Cognition: A Recommended Approach

Developing targeted interventions to ameliorate deficits in ToM and other social cognitive functions represents a promising transdiagnostic treatment avenue for enhancing wellbeing in clinical populations. Effectively treating these impairments requires understanding the large-scale network interactions underlying social cognition [4], identifying the causal contributions of specific brain regions [24], and employing behavioral assessments capable of detecting and isolating social cognitive deficits [70]. Measuring network interactions with anatomical precision requires techniques with high spatiotemporal resolution, i.e., an ability to accurately assess where and when neural activity is occurring. Identifying causal mechanisms of brain function necessitates methods that can directly manipulate neural activity with transient effects. Equally critical are behavioral assessments that can reliably distinguish social cognition from related domains (specificity) and detect impairments in clinical populations (sensitivity). Integrating iEEG and iES with psychometrically sound measures offers a promising approach for advancing both scientific understanding of social cognition and the development of clinically meaningful interventions. Below, we outline key recommendations to guide future studies in this emerging research area.

### Selection of Regions of Interest and Social Cognitive Assessments

Research using fMRI has contributed the most to our understanding of how the brain supports social cognition by localizing the distributed neural networks involved in these processes. Therefore, when designing iEEG/iES paradigms to assess social cognition, researchers should leverage the extensive fMRI literature to guide their decisions. Prior fMRI findings can be used to guide the selection of behavioral tasks and regions of interest in iEEG or stimulation targets in iES. This ensures the social cognitive function probed by the task is linked to the brain areas being measured, increasing the interpretability and translational value of neural finding [96]. Moreover, tasks should demonstrate clinical specificity—that is, they must be capable of isolating social cognitive processes from other cognitive domains such as language, memory, or executive function. This is particularly important when working with clinical populations, where comorbid deficits are common and may confound interpretation. Employing tasks with demonstrated sensitivity to social cognitive impairments in focal epilepsy (e.g., the RMET) or related other disorders enhances the clinical relevance of the findings and strengthens the potential for identifying meaningful brain–behavior relationships.

### Consideration of Social Cognitive Deficits

Most patients with DRE will be diagnosed with TLE or FLE [25], in which patients often experience social cognitive impairment, particularly in ToM and emotion recognition [1, 2, 88, 91, 97]. Therefore, it is likely that iEEG/iES studies of social cognition will contain a subset of participants with deficits in these processes. However, despite the body of work linking social cognitive dysfunction to focal epilepsy, these methods rarely consider or assess patients’ social cognitive ability to determine whether deficits exist. Assessing social cognition prior to task performance will enable researchers to account for individual variability and more accurately interpret neural findings. Importantly, although many patients may show intact abilities, identifying those with impairments offers a valuable opportunity to investigate the neural mechanisms underlying social cognitive dysfunction, ultimately advancing both basic science and clinical translation.

### Analysis of Task-Based Connectivity Metrics

Social cognition relies on coordinated activity across widely distributed brain regions. To move beyond localization and characterize how these regions coordinate during social processing, task-based iEEG and iES studies should incorporate connectivity analyses. In passive iEEG paradigms, functional connectivity methods, such as coherence or phase-locking, can identify synchronous activity between regions engaged by the task, while effective connectivity approaches, such as spectral Granger causality, can reveal directional influences among nodes in the functional network [32]. Complementarily, iES can assess how direct perturbation of brain regions affects measures of functional and effectivity connectivity between critical sites and other nodes by simultaneously collecting iEEG recordings from other electrodes located within the network to assess causal network relationships. Similarly, iES can be delivered while simultaneously collecting whole-brain fMRI data. Recently, a framework was proposed for combining these techniques to identify causal interactions within the networks underlying emotion recognition and other affective processes [98]. In an effort to assess amygdala connectivity, researchers applied iES to the amygdala while simultaneously collecting whole-brain resting-state fMRI data in four neurosurgical patients with DRE [98]. To increase the generalizability of their results, they established a baseline causal network using large-scale, resting-state datasets from healthy individuals (i.e., the Human Connectome Project [99] and MyConnectome Project [100]) and verified that patients’ resting-state connectivity patterns were similar [98]. Their results showed stimulating the amygdala reliably evoked responses in nodes commonly associated with emotion recognition, including the insula, dorsomedial prefrontal cortex, cingulate cortex, and superior temporal gyrus [98]. As the authors highlight, this integrated approach holds promising clinical applications, including generating hypotheses about the effect of neuromodulation therapies, such as deep brain stimulation (DBS), for the treatment of mood and anxiety disorder [98].

## Future Directions

### Understanding Social Cognitive Deficits in Epilepsy

Epilepsy patients undergoing surgery evaluation with intracranial electrodes represent a particularly valuable population for studying social cognition, as many undergo implantation in frontal and temporal regions that overlap with core social brain networks, and the presence of both intact and impaired social cognitive abilities across patients provides a broad spectrum of functioning to investigate. Although much of the iEEG and iES research in cognitive neuroscience aims to produce generalizable insights into normative brain function, future work should also capitalize on the unique opportunity these methods offer to investigate the neural mechanisms underlying social cognitive deficits in individuals with focal epilepsy. Neuroimaging studies have revealed abnormal functional connectivity during social cognitive tasks in this population, suggesting that network-level disruptions may contribute to impaired performance [101–103]. This view aligns with the latest perspective of epilepsy as a systems-level disorder, arising from the disruption of distributed networks, rather than discrete brain regions [104, 105]. However, most of the research investigating the neurobiology of these deficits has focused on determining the impact of the SOZ on task performance, with limited studies exploring dysfunction in the rest of the network [106]. By leveraging the high spatial and temporal precision of iEEG and the causal perturbation afforded by iES, researchers can directly test how altered connectivity or circuit dynamics in key social brain regions relate to specific behavioral deficits. These findings can also clarify whether network dysfunction linked to social cognitive deficits in epilepsy (e.g., the relationship between diminished amygdala connectivity and ToM performance [101]) is causative, compensatory, or secondary to other neural disruptions. Such an approach not only extends the translational relevance of this work but also holds promise for identifying network-based biomarkers or intervention targets for patients with social cognitive impairment.

## Conclusion

Social cognition is gaining recognition as a clinically important cognitive domain, with growing advocacy for evaluating processes like ToM and emotion recognition in clinical settings [11, 16, 17, 70–73]. Research shows that social cognitive dysfunction is common across most neuropsychiatric conditions, including epilepsy, and can significantly diminish QoL [2–4, 10, 11, 70, 76, 107, 108]. These deficits are now seen as arising from abnormal interactions within social brain networks rather than isolated region-specific disruptions [4]. Improving social cognition is a promising transdiagnostic treatment avenue, but developing targeted therapies requires better understanding of social brain network dynamics. However, the prevailing technique in human brain mapping research, fMRI, is not capable of measuring neural activity at the millisecond scale of cognition or pinpointing brain regions that cause a symptom or behavior, limiting the identification of neuromodulatory treatment targets for brain disorders. IEEG and iES are techniques that can address these gaps with high spatiotemporal resolution and ability to manipulate brain function with focal stimulation. We recommend an approach that integrates iEEG and iES with well-validated behavioral assessments that demonstrate both clinical sensitivity and specificity. This combination allows researchers to precisely localize and manipulate neural activity underlying social cognition, while ensuring that observed effects are meaningfully tied to behavior. To enhance the translational impact of iEEG and iES studies of social cognition, we recommend several best practices. First, tasks and regions of interest should be grounded in prior fMRI research and selected for their ability to isolate social cognitive processes with clinical specificity. Second, behavioral assessments must be psychometrically validated to ensure sensitivity to deficits in relevant patient populations. Finally, task-based connectivity analyses—both functional and effective—should be used to capture the dynamic interactions among brain regions with particular attention to disruptions that may underlie social cognitive impairments in focal epilepsy. Together, these strategies will improve the precision, interpretability, and clinical relevance of research in this emerging field.

## Key References

- Tan, K. M., Daitch, A. L., Pinheiro-Chagas, P., Fox, K. C. R., Parvizi, J., & Lieberman, M. D. (2022). Electrocorticographic evidence of a common neurocognitive sequence for mentalizing about the self and others. *Nature Communications*, *13*(1). 10.1038/s41467-022-29510-2.
  - ⚬ iEEG study examining the spatiotemporal sequence of ToM, findings show a pattern of activation that begins in the visual cortex, progresses through temporo-parietal areas, and ends in medial prefrontal regions and revealed later-stage regions exhibit greater functional specificity and thinking about others vs oneself evoked slower more prolonged activation, suggesting longer processing.

- Yordanova, Y. N., Duffau, H., & Herbet, G. (2017). Neural pathways subserving face-based mentalizing. *Brain Structure and Function*, *222*(7), 3087–3105. 10.1007/s00429-017-1388-0
  - ⚬ iES study that identified cortical and subcortical regions critical for ToM, revealing that the ability to infer mental states from facial cues depends on the functional integrity of both the inferior fronto-occipital fasciculus and the superior longitudinal/arcuate fasciculus, highlighting a ventral and dorsal white matter network supporting social cognition.

- S. H. Siddiqi, K. P. Kording, J. Parvizi, and M. D. Fox, “Causal mapping of human brain function,” Jun. 01, 2022, *Nature Research*. 10.1038/s41583-022-00583-8.
  - ⚬ Review describing iES and other causal brain mapping techniques and where they fall on a proposed continuum of causality and how these techniques can be combined with correlative neuroimaging methods to guide the development of targeted treatments for brain disorders.

- J. Parvizi and S. Kastner, “Promises and limitations of human intracranial electroencephalography,” Apr. 01, 2018, *Nature Publishing Group*. 10.1038/s41593-018-0108-2.
  - ⚬ Review outlining the strengths and limitations of iEEG for exploring human brain function with some discussion of iES.

- Cotter, J., Granger, K., Backx, R., Hobbs, M., Looi, C. Y., & Barnett, J. H. (2018). Social cognitive dysfunction as a clinical marker: A systematic review of meta-analyses across 30 clinical conditions. In *Neuroscience and Biobehavioral Reviews* (Vol. 84, pp. 92–99). Elsevier Ltd. 10.1016/j.neubiorev.2017.11.014.
  - ⚬ Systematic review of meta-analyses conducted across 30 different neuropsychiatric conditions, including epilepsy, that investigated social cognitive functioning, revealed deficits in ToM and facial emotion recognition for all disorders and suggested these impairments may serve as a general marker of brain dysfunction

## Supplementary Information

Below is the link to the electronic supplementary material.

Supplementary file1 (PDF 1.19 MB)

Supplementary file2 (PDF 1.19 MB)

Supplementary file3 (PDF 1.74 MB)

## Data Availability

No datasets were generated or analysed during the current study.
